# Negative pressure wound therapy for complex oral wounds

**DOI:** 10.1093/jscr/rjad638

**Published:** 2023-12-06

**Authors:** Mykaihla Sternick, James C Gates, Allen Champion, Andrew Yampolsky

**Affiliations:** Sidney Kimmel Medical College, Thomas Jefferson University, 909 Walnut Street, Philadelphia, PA 19106, United States; Department of Oral and Maxillofacial Surgery, Hospital of the University of Pennsylvania, Penn Medicine, Philadelphia, PA 19104, United States; Department of Oral and Maxillofacial Surgery, Sidney Kimmel Medical College, Thomas Jefferson University, 909 Walnut Street, Philadelphia, PA 19106, United States; Department of Oral and Maxillofacial Surgery, Sidney Kimmel Medical College, Thomas Jefferson University, 909 Walnut Street, Philadelphia, PA 19106, United States

**Keywords:** complex oral wounds, negative pressure wound therapy, orocutaneous fistula, oncology, reconstruction

## Abstract

Negative pressure wound therapy provides a nonsurgical treatment option for many types of complex wounds. This therapy utilizes the vacuum-assisted closure device, which decreases air pressure, removes fluid that accumulates within the wound, and aids to facilitate changes that promote healing. Despite the increased use of negative pressure wound therapy in the head and neck region, there is substantially less data available on the management of transoral vacuum-assisted wound closure. Herein, we present a case of a novel approach for the creation and use of a transoral wound vac for a patient with a refractory orocutaneous wound in the setting of multiple previous free flaps and surgeries. A watertight seal was able to be maintained and the patient was compliant with treatment, resulting in successful management of the wound. We promote the consideration of this novel technique’s use for similar difficult-to-treat oral wounds.

## Introduction

“Negative pressure wound therapy” (NPWT) has provided a nonsurgical form of treatment for many types of complex wounds. This therapy utilizes the “vacuum-assisted closure device” (VAC), characterized by Argenta and Morykwas [1]. The device consists of a semi-occlusive dressing overlying a polyurethane foam sponge, which is placed into the wound. This sealed wound is attached to a tube connected to a pump, which supplies a suction force, typically of 125 mmHg, to the wound [2]. The VAC decreases dead space and its associated fluid collection, subsequently decreasing tissue edema and aiding facilitation of macroscopic and microscopic changes that promote healing [2]. The polyurethane foam induces angiogenesis, thus increasing the delivery of blood, oxygen, and nutrients that are critical to healing, whereas the induction of negative pressure leads to stimulation of cell proliferation and growth factors, promoting new tissue growth. In addition, the negative pressure aids in approximating the wound edges [2]. It is hypothesized that the withdrawal of this tissue edema combined with the promotion of new, vascularized tissue growth helps to reverse the hypoxia of these fluid rich dead spaces, leading to more rapid and definitive healing. NPWT has been detailed for management in the head and neck region to treat necrotizing fasciitis, open facial fracture wounds, management of fistulas following osteoradionecrosis and reconstruction, and others [3–5].

Similarly, management of transoral wounds is often quite arduous, as reconstructive options may be exhausted, or the patient may no longer be a candidate for surgical treatment. We present a case of the use of NWPT for the successful treatment of a complex, full-thickness oral wound and promote its consideration of use for similar difficult-to-treat oral wounds.

## Methods

The patient was a 73-year-old female diagnosed with squamous cell carcinoma of the left buccal mucosa with mandibular invasion. She underwent primary resection, which included mandibulectomy and reconstruction with a parascapular free flap. Initially, she did well both oncologically and functionally. However, she developed osteoradionecrosis with pathologic fracture and an associated orocutaneous fistula at the treated site ~10 years following her original treatment. The patient then underwent a segmental resection and reconstruction with an osteocutaneous fibula free flap in that same site.

**Figure 1:**
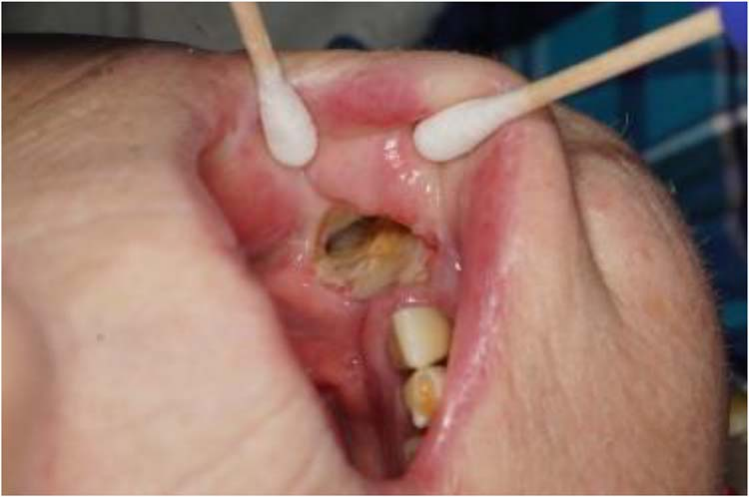
Photograph showing the full thickness intraoral component of the orocutaneous fistula with exposed fibula bone.

**Figure 2:**
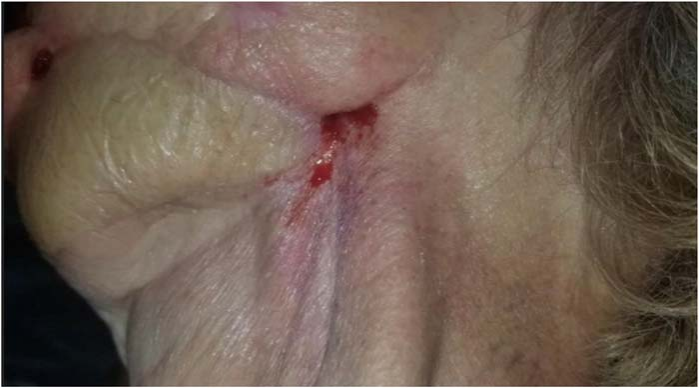
Photograph showing the cutaneous component of the orocutaneous fistula. Also, note significant scar contracture and evidence of radiation fibrosis of the skin.

Following this surgery, she was discharged on post op day 6 after an uneventful postoperative course. At the 1-week follow-up, the native oral mucosa overlying the fibula flap had broken down, and a significant orocutaneous fistula with resultant salivary leak developed (Figs 1 and 2). There was concern of anastomotic compromise because of the proximity of the salivary collection to the pedicle, and potential for resultant flap loss. Extensive remobilization of the existing inset or elevation of a soft tissue flap would be necessary for surgical salvage. After much deliberation of options, the decision was made to undertake a novel, nonsurgical treatment option: transoral NPWT.

**Figure 3:**
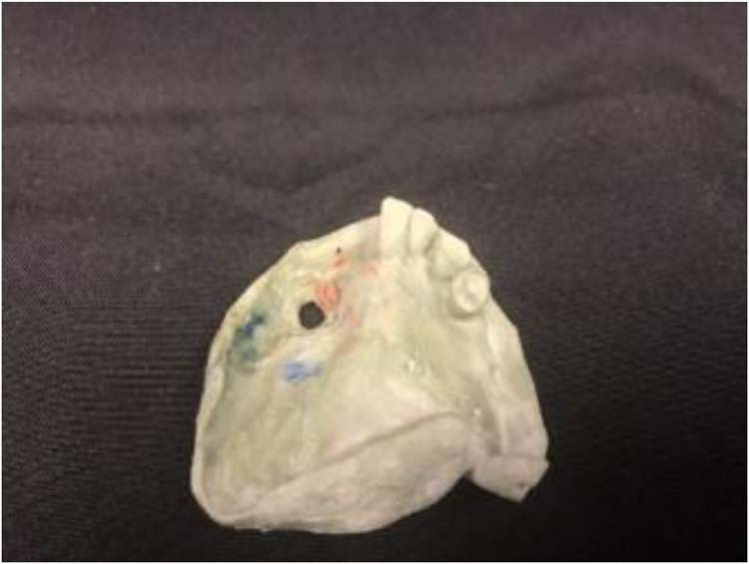
Stone model for the intraoral device with full thickness defect noted.

**Figure 4:**
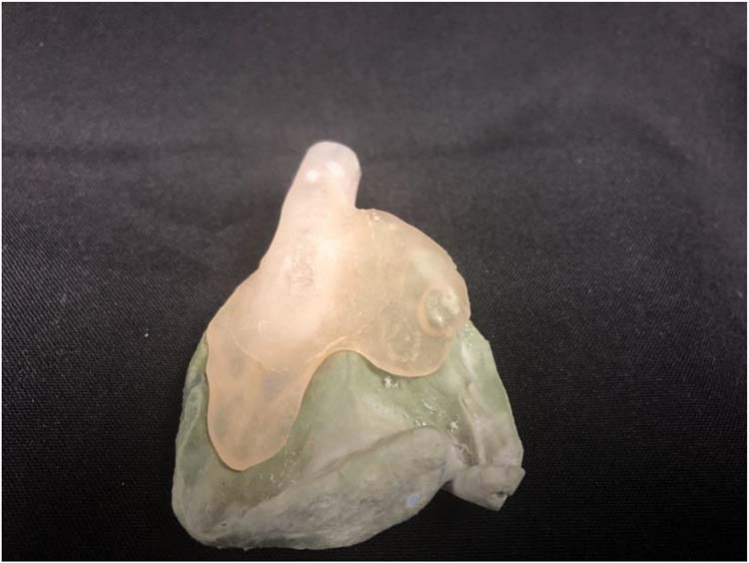
Custom tray overlying stone model prior to relining with dental impression material.

**Figure 5:**
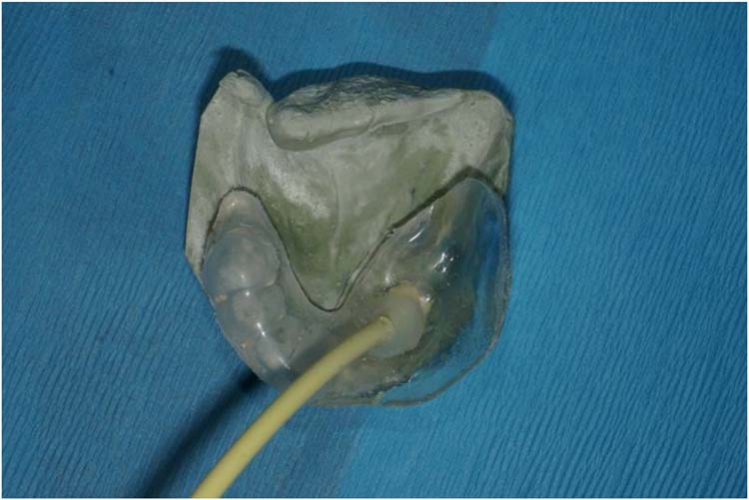
Acrylic splint with perforated area attached to tubing to attach to VAC system overlying the stone cast as it would then eventually sit within the oral cavity.

In order to fabricate a custom oral VAC device, dental impressions were obtained from the patient, and a stone model was poured to create a template on which to fashion a custom tray (Fig. 3). The custom tray was fabricated and verified using the stone model (Fig. 4). This tray was then relined in the mouth with polyvinyl siloxane impression material to ensure adequate seal against the mucosa and this impression was then used to fabricate the acrylic splint to overly the defect. This splint was then perforated and connected to a VAC device and inserted into the oral cavity overlying a standard polyurethane foam that was inserted into the wound (Fig. 5). A polyurethane foam VAC sponge was adapted to the cutaneous area and a Y-connector was placed to apply negative pressure to both sites simultaneously.

**Figure 6:**
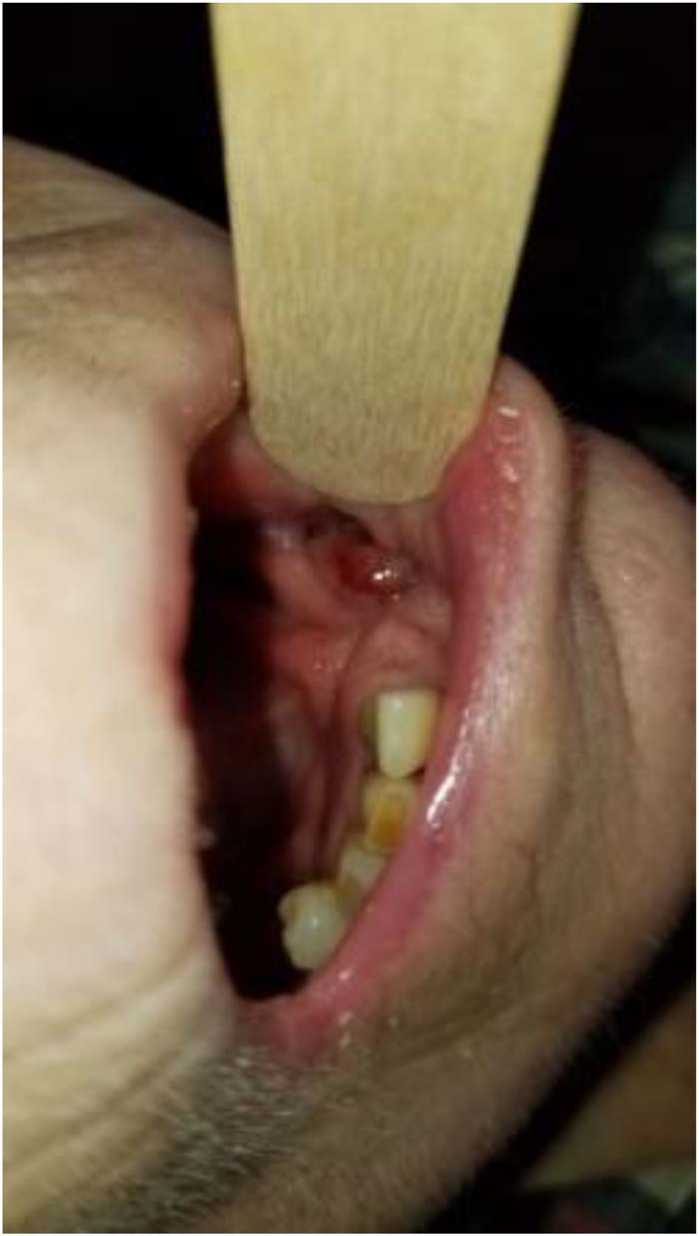
There is near complete mucosalization of the oral defect 2 weeks postinitiation of combined transoral and transcervical NPWT.

**Figure 7:**
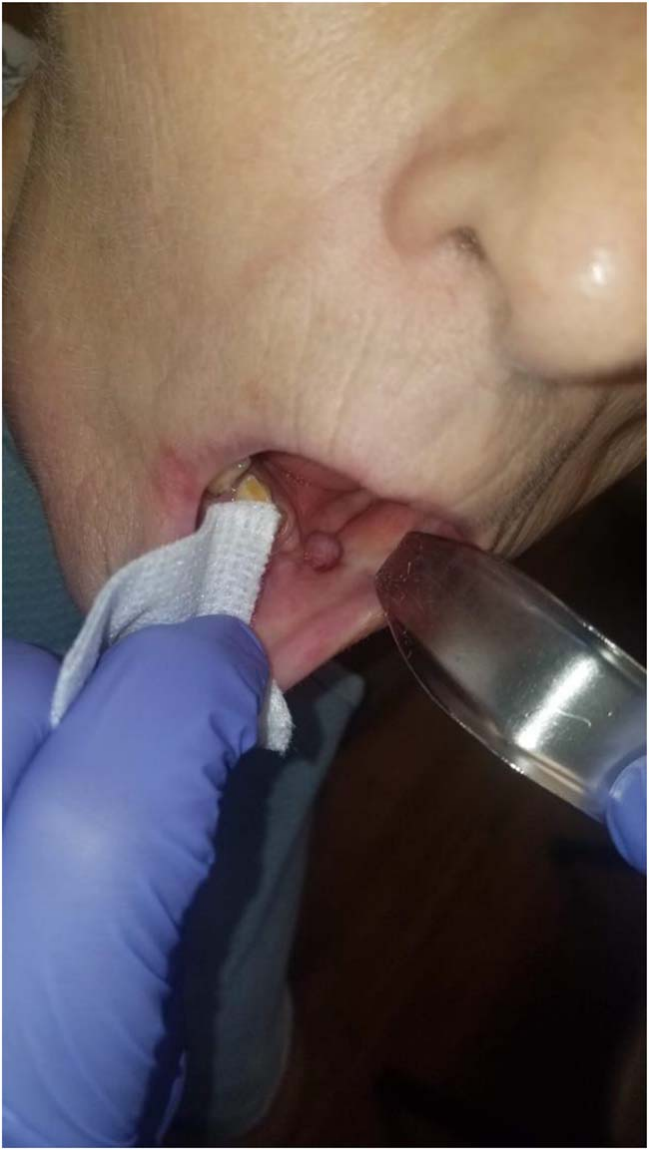
There is mucosalization of the oral defect and diminished erythema and edema of the healing wound as compared with Fig. 6 2 weeks prior.

The device was well tolerated and worn continuously for at least 18 hours a day, with short periods allowed to disconnect; it maintained successful, continuous suction. Every third day, the VAC dressing and sponge were changed, inserting a smaller sized sponge. The patient was fed via a Dobhoff tube during this time. Two weeks later, the patient showed granulation tissue formation in her intraoral wound with almost complete closure (Fig. 6). At this point, the intraoral device was discontinued, whereas the VAC dressing on the neck was maintained for two additional weeks until the intraoral component of the fistula had mucosalized completely and was no longer able to be probed (Fig. 7).

## Discussion

In this report, we aimed to examine the efficacy and feasibility of a novel treatment for a complex oral wound in a multiply operated patient. We found that the custom tray facilitated transoral wound NPWT, afforded significant formation of healing granulation tissue over the 2 weeks following the initiation of treatment, and helped avoid additional surgery in a multiply operated patient.

Multiple studies have previously demonstrated the success of using VAC for orocutaneous and pharyngocutaneous fistulas, using an external cutaneous approach [6–8]. One approach included watertight suturing of the mucosal side of the wound, with VAC placement in the dead space of the cutaneous side. Four of six healed via VAC assisted secondary intention [8]. Additional approaches included attempts to use a tissue sealant for the mucosal side of orocutaneous fistulas, with the VAC device placed on the cutaneous component [9, 10].

In the only study discoverable which used intraoral wound vac, three patients with medication-related osteonecrosis of the jaw were treated via a similar intraoral approach as in this report. They showed increased granulation tissue and pain relief while citing no serious negative effects of the oral VAC therapy. These patients only required an intraoral suctioning device [11].

The limitations of this treatment modality include feasibility and tolerance of wearing a device intraorally. Because of the mucosal environment, difficulties persist to keep a water and airtight seal, leading to the sustained suction forces necessary for the VAC devices. Exceptional patient compliance is necessary for these reasons, as well as frequent follow-up visits. We advocate for further pooling and publication of institutional data that might further validate its use.
